# Boosting Male Fertility: The Impact of Gonadotropin Therapy on Hypogonadotropic Hypogonadism—A Systematic Review and Meta‐Analysis

**DOI:** 10.1111/andr.70226

**Published:** 2026-04-16

**Authors:** Manou Huijben, Anne Prinsen, Laetitia de Kort, Jetske van Breda

**Affiliations:** ^1^ Department of Urology University Medical Centre Utrecht Utrecht the Netherlands

**Keywords:** azoospermia, gonadotropins, male fertility, male hypogonadotropic hypogonadism, pregnancy, spermatogenesis

## Abstract

**Background:**

Male hypogonadotropic hypogonadism typically presents with azoospermia and is one of the few causes of infertility amenable to a medical intervention. Gonadotropin therapy offers a chance to restore spermatogenesis and fertility in these individuals. Therefore, this study aims to determine the efficacy of gonadotropins in inducing spermatogenesis and achieving pregnancies in males with hypogonadotropic hypogonadism and azoospermia. Additionally, the time needed to induce spermatogenesis and pregnancy, and potential predictors associated with better outcomes, were evaluated.

**Methods:**

A systematic literature search was conducted in the Medline, Embase, and Cochrane databases till May 16, 2025. Clinical studies, including randomized control trials, cohort studies, and case‐control studies, using gonadotropins as a treatment for hypogonadotropic hypogonadism and assessing sperm induction or pregnancy outcomes, were eligible for inclusion. Primary outcomes included sperm parameters (appearance, concentration, motility, morphology, time needed to induce spermatogenesis) and pregnancy outcomes (pregnancy rate, proportion of naturally achieved pregnancies, proportion of live births, and time to conception). The methodological quality and risk of bias were assessed using the Effective Public Health Practice Project quality assessment tool for quantitative studies, and showed that most included studies were of poor methodological quality.

**Results:**

A total of 50 studies were included from 2351 identified articles, treating 1583 male patients with hypogonadotropic hypogonadism and azoospermia. The pooled success rate of spermatogenesis induction was 74% (95% confidence interval: 66%–82%), with a mean time to spermatogenesis of 11.8 months. The overall pregnancy rate among couples with a child wish was 52% (95% confidence interval: 42%–62%), with a mean time to pregnancy from the start of treatment of 19 months. Mean post‐treatment sperm concentration was 9.8 million/mL (95% confidence interval 8.6–11.0), and spontaneous pregnancies also occurred with suboptimal sperm parameters. Gonadotropin dual therapy was more effective than human chorionic gonadotropin monotherapy.

**Conclusion:**

Gonadotropin therapy, particularly combined gonadotropin treatment, is effective in restoring spermatogenesis and achieving pregnancy in men with hypogonadotropic hypogonadism. While sperm quality often remains below normal thresholds, natural conception is frequently achieved. These findings support the use of combined gonadotropin therapy as the first‐line approach for fertility induction in this population.

**Trial Registration**: This review was registered on PROSPERO under the registration number CRD4202232149. A review protocol was not previously published.

## 1 Introduction

Hypogonadotropic hypogonadism (HH) is a condition characterized by insufficient secretion of gonadotropins, luteinizing hormone (LH), and follicle‐stimulating hormone (FSH) by the pituitary gland [1]. In men, this results in disturbed testicular function, with reduced testosterone production and absent or severely impaired spermatogenesis. This could cause symptoms such as delayed puberty, decreased libido, erectile dysfunction, fatigue, muscle weakness, and infertility [1, 2]. HH can arise from congenital causes, e.g., Kallmann syndrome, or acquired conditions, e.g., hyperprolactinemia, obesity, and pituitary neoplasms [3].

Fertility treatment options for men with HH include selective estrogen receptor stimulators (SERMs) such as clomiphene citrate, gonadotropin‐releasing hormone (GnRH) therapy, and gonadotropin‐replacement therapy using recombinant or human chorionic gonadotropin (hCG) and FSH [4]. GnRH therapy and gonadotropin therapy have comparable fertility outcomes, though GnRH therapy is accompanied by high costs, is less available, and is only effective in patients with preserved pituitary function [5, 6, 7]. A less costly and non‐invasive treatment option is clomiphene citrate, in daily practice considered as a first‐line option in idiopathic HH [8, 9]. However, according to the European Association of Urology Guideline Sexual and Reproductive Health, gonadotropin therapy is the standard treatment for men with HH and impaired fertility [2]. hCG therapy simulates LH and stimulates Leydig cells in the testes to produce testosterone, while FSH therapy stimulates Sertoli cells, increasing spermatogenesis [10]. Combination therapy (LH and FSH) is based on the synergistic roles of LH (mimicked by hCG) and FSH in testicular function [11, 12]. Nevertheless, evidence regarding the timing and added benefit of FSH compared with hCG monotherapy remains limited and heterogeneous, underscoring the need for further investigation [12, 13].

Previous studies have shown that male HH is one of the few causes of infertility that can be treated with gonadotropin therapy [7, 14]. A systematic review and meta‐analysis on this topic was done in 2014, but did not assess the time required for spermatogenesis induction and predictive parameters [15]. The time required to induce spermatogenesis and pregnancy, and the determinants of treatment success, have not been sufficiently characterized.

Therefore, the aim of this systematic review and meta‐analysis is to give an update on the currently available literature on the effects of gonadotropins in males with HH in terms of spermatogenesis, time needed to induce spermatogenesis, achievement of pregnancies, and predictive values for treatment success.

## 2 Methods

### 2.1 Search Strategy

This systematic review and meta‐analysis adhered to the guidelines of Preferred Reporting Items for Systematic Reviews and Meta‐Analyses [16]. The study was registered in PROSPERO under registration number CRD4202232149. The electronic databases Medline (1946–May 16, 2025), EMBASE (1947–May 16, 2025), and Cochrane Central Register of Controlled Trials (1992‐ May 16, 2025) were systematically searched. Search terms covered the study domains ‘hypogonadotropic hypogonadism’, the intervention ‘gonadotropins’, and outcomes related to ‘spermatogenesis’ and ‘pregnancy’. The full search strategy is provided in Table S1. Filters were applied to restrict results to human studies published in English. Additionally, citation screening of articles selected for full‐text review was performed to identify additional relevant studies.

### 2.2 Selection Process

Two independent authors (A. M. A. Prinsen and M. Huijben) independently screened titles and abstracts for eligibility. Subsequently, full‐text articles of potentially relevant studies were evaluated for inclusion. Disagreements were resolved by discussion and consensus. The eligibility criteria required the inclusion of adult men with HH (total testosterone <12 nmol/L in combination with low FSH and LH who were treated with gonadotropin therapy, and who were evaluated for spermatogenesis or who achieved pregnancy through either clinical intervention or observational study. Studies were excluded if they involved women, animals, in vitro settings, or alternative fertility treatments (e.g., pulsatile GnRH, SERMs, and aromatase inhibitors), or if gonadotropin therapy was combined with other agents (e.g., testosterone, SERMS, and GnRH). Studies evaluating pulsatile GnRH therapy were excluded because this treatment requires intact pituitary function and specialized administration, making it less comparable to gonadotropin replacement therapy in clinical practice. Excluding these studies allowed a more homogeneous analysis of gonadotropin‐based interventions. Additional exclusion criteria included narrative reviews, case reports, case series (*n* < 5), conference abstracts, editorials, and publications not available in full text or not published in English. In cases where multiple publications reported data from the same or overlapping cohorts, the study with the most comprehensive data set was included.

### 2.3 Data Extraction

Data collection was independently conducted by two reviewers (A. M. A. Prinsen and M. Huijben) using a standardized data extraction form. Extracted items including, study design, sample size, patient characteristics (e.g., age, etiology of HH, percentage pre‐pubertal HH onset of HH, previous puberty induction, prevalence of cryptorchidism [uni‐, or bilateral], baseline testicular volume), gonadotropin therapy characteristics (e.g., type of gonadotropin(s), dosage, and duration of therapy), and outcomes of interest. Primary outcomes included sperm parameters (appearance, concentration, and time needed to induce spermatogenesis) and pregnancy outcomes (pregnancy rate, proportion of naturally achieved pregnancies, proportion of live births, and time to conception). Spontaneous pregnancy was defined as a pregnancy occurring without any medical intervention, such as assisted reproductive techniques, including intrauterine insemination, in vitro fertilization, or intracytoplasmic sperm injection. Missing values or outcomes that could not be derived from text or figures were reported as “not specified”. Analyses were performed using the available sample size and reported statistics only, without data imputation.

### 2.4 Quality Assessment

The methodological quality and risk of bias of each included study were assessed independently by two reviewers (A. M. A. Prinsen and M. Huijben), using the Effective Public Health Practice Project (EPHPP) quality assessment tool for quantitative studies [17]. This tool allows comparison of studies with various study designs and has been previously validated for use in systematic reviews of effectiveness [18]. Domains assessed included selection bias, study design, confounding, blinding, data collection methods, and withdrawals/dropouts. The EPHPP tool has demonstrated excellent inter‐rater reliability, with an inter‐assessor coefficient of 0.77 (95% confidence interval [CI], 0.51–0.90). Each study received a global rating of ‘strong’, ‘moderate’, or ‘weak’. Disagreements were resolved by consensus.

### 2.5 Data Analysis

Outcomes were presented as frequencies with percentages for categorical variables and as means with standard deviations (SDs) for continuous variables. These data were calculated if studies reported outcomes separately for each patient enrolled. Cochrane's formula was used to combine means and SDs of subgroups if data were not reported separately [19]. Outcomes reported by five or more studies were pooled in a meta‐analysis conducted using R software (R Foundation for Statistical Computing, Vienna, Austria).

Studies reporting dichotomous outcomes were eligible for meta‐analysis if proportion data were available. The cumulative event rate, representing the estimated occurrence of the outcome at one time point, was used to pool the data, with 95% confidence interval as the effect size. A logit transformation was applied to stabilize variance and handle extreme values near 0 or 1. For continuous outcomes, pooled results were expressed as mean values with 95% CI. If studies reported medians with interquartile ranges or minimum to maximum values, these were converted to means with SDs using established formulas, provided the data distribution was not significantly skewed [20, 21, 22].

Heterogeneity among studies was assessed visually through forest plots and statistically by the *I* ^2^ statistic. The interpretation of *I* ^2^ was as follows: 0%–40% may not be important, 30%–60% may represent moderate heterogeneity; 50%–90% may represent substantial heterogeneity, and 75%–100% may represent considerable heterogeneity [23]. As substantial heterogeneity was evident in all intended meta‐analyses, the DerSimonian‐Laird random effects estimator was employed to calculate effect sizes [23]. Publication bias was assessed using funnel plots and Egger's regression test for asymmetry for key clinical outcomes (sperm appearance, sperm concentration, time to spermatogenesis, and pregnancy success rate).

### 2.6 Subgroup and Sensitivity Analyses

Subgroup analyses were performed based on clinical variables including onset of HH (pre‐ vs. post‐pubertal), type of gonadotropin therapy, timing of FSH initiation, prior testosterone replacement therapy (TRT), and presence of cryptorchidism. Subgroup analyses were conducted only when at least five studies reported comparable outcomes, to ensure adequate statistical power and reduce small‐study effects. In addition, meta‑regression analyses were performed to examine whether mean testicular volume was associated with key treatment outcomes, including spermatogenesis success rate, sperm concentration, and the time required to achieve spermatogenesis. Sensitivity analysis was conducted by restricting analyses to studies exclusively including adults (≥18 years) and those explicitly reporting azoospermia as an inclusion criterion.

## 3 Results

### 3.1 Study Selection

**FIGURE 1:**
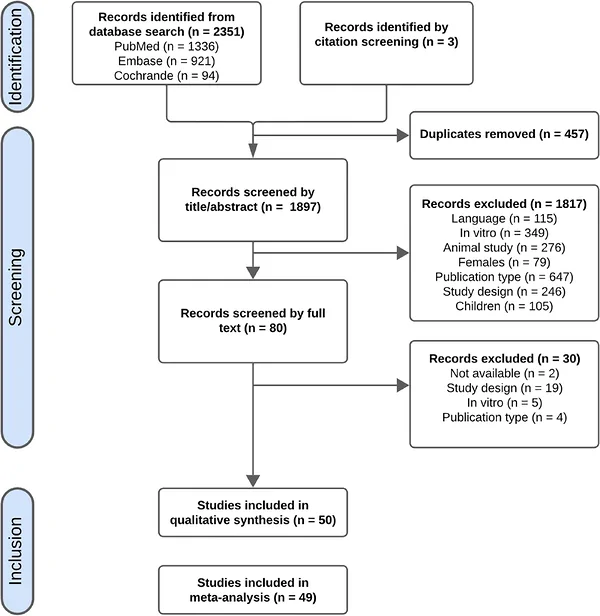
PRISMA (Preferred Reporting Items for Systematic Reviews and Meta‐Analyses) flow chart of search and study selection.

The initial database search yielded 2351 records, with an additional three records identified through manual citation screening. After removal of duplicates, 1897 records remained (Figure 1). After screening for relevance by title and abstract, 80 full‐text articles were assessed for eligibility. Finally, 50 included articles were critically appraised on their risk of bias. Reasons for exclusion are displayed in Figure 1.

### 3.2 Study Characteristics

In total, 50 studies (21 prospective intervention studies, 20 retrospective cohort studies, and nine with unclear study design) treating 1583 male patients with HH and azoospermia were included (Table 1). Several studies reported outcomes per subgroup, such as those who received gonadotropins, prior TRT, or onset of HH, and were included in the meta‐analysis accordingly. The mean age of patients undergoing gonadotropin‐replacement therapy ranged from 20 to 44 years, including one study focusing on older men with idiopathic HH. Mean testicular volume, as measured by a Prader orchidometer, varied between 1.6 and 10.5 mL. The prevalence of cryptorchidism ranged from 0% to 44% across 27 studies. In six studies, only unilateral cases were reported; one study reported bilateral cases, one study included a mixed population, and 19 studies did not specify the type of cryptorchidism (Table 1). Only 18 out of 38 studies, including pre‐pubertal onset of HH, reported on puberty induction, without analyzing subgroups (Table 1). Mean therapy or follow‐up duration varied between 4 and 85 months.

**TABLE 1:** Study characteristics.

First author	Year	Study type	Country	Sample size	Age, years (mean ± SD or range)	Testis volume, ml (mean ± SD or range)	Cryptorchidism (%) (unilateral, bilateral, or n.s.)	Pre‐pubertal onset HH (%)	Previous puberty induction (%)	Previous TRT (%)	Gonadotropin replacement	FSH onset after/simultaneous hCG/varied	When FSH addition	Dosage of gonadotropins	Duration of therapy, months (mean ± SD or range)	Follow‐up, months	Global EPHPP rating
Abbasi [24]	2008	Non‐comparative intervention study	Iran	56	31 ± 6	n.s.	n.s.	100	n.s.	n.s.	hCG + FSH	After	n.s.	hCG 5000 IU 3x/week	27	n.s.	Weak
Bakircioglu [25]	2007	Non‐comparative intervention study	Turkey	25	34 ± 5	3.7 ± 1.9	4 (unilateral)	n.s.	n.s.	40	hCG + FSH	After	1 month	hCG 5000 IU 2x/week	n.s.	n.s.	Weak
Bouloux [26]	2002	Non‐comparative intervention study	Europe	19	n.s.	2.5 ± 1.0	n.s.	100	n.s.	n.s.	hCG + FSH	After	2 months	hCG 2000 2x/week, adjusted to TT level. FSH 150 IU 3x/week	18	24	Weak
Bouloux [27]	2003	RCT	Australia	30	30 ± 7	4.8 ± 2.8	37 (n.s.)	n.s.	n.s.	n.s.	hCG + FSH	After	n.s.	hCG 1500 IU 2x/week	11	n.s.	Weak
Burgues [28]	1997	Non‐comparative intervention study	Spain	60	26 ± 1	4.3 ± 3.9	0	87	82	78	hCG + FSH	After	1 month	hCG 2500 IU 2x/week	1	n.s.	Weak
Burris [29]	1988	n.s.	USA	20	24 ± 4	4.6 ± 3.6	5 (unilateral)	100	n.s.	90	hCG	n.a.	n.a.	hCG 2000 IU 3x/week	40 ± 33	n.s.	Weak
Carani [30]	1999	Non‐comparative intervention study	Italy	7	30 ± 3	n.s.	n.s.	n.s.	n.s.	43	hCG + FSH	Simul	n.a.	hCG 2000 IU 2x/week	4	4	Weak
Chen [31]	2020	Retrospective cohort	China	110	21 ± 5	n.s.	12 (n.s.)	n.s.	n.s.	n.s.	hCG	n.a.	n.a.	hCG 2000 IU 2x/week, adjusted to TT levels	n.s.	40	Weak
Chen [32]	2021	Retrospective cohort	Taiwan	17	36 ± 4	5.8 ± 4.1	n.s.	n.s.	n.s.	53	Varies	After	Persistent azoospermia	hCG 3000 IU/week, adjusted to TT levels	11	n.s.	Weak
Efesoy [33]	2009	Non‐comparative intervention study	Turkey	21	23 ± 8	4.1 ± 0.6	n.s.	n.s.	n.s.	n.s.	hCG + FSH	After	normal T level	hCG 1500 IU 2–3x/week	14 ± 5	n.s.	Weak
European Metrodin HP Study Group [34]	1998	Non‐comparative intervention study	France	19	23 ± 7	3.5 ± 1.7	0	100	52	42	hCG + FSH	After	6 months of azoospermia	hCG 2000 IU 2x/week, adjusted to TT levels	3‐6	At least 21	Moderate
Finkel, [35] Post‐pub Pre‐pub	1985	Non‐comparative intervention study	USA	5 13	39 ± 12 29 ± 4	n.s. n.s.	0 0	0 100	n.s. n.s.	n.s. n.s.	hCG hCG + FSH	n.a. After	n.a. n.s.	hCG 2000 IU 3x/week, adjusted to TT levels	n.s. n.s.	n.s. n.s.	Weak
Fuse, [36] hCG hCG +hMG	1996	Retrospective cohort	Japan	8 8	24 ± 6 25 ± 4	2.7 ± 1.9 4.9 ± 4.8	25 (n.s.) 0	100 100	n.s. n.s.	n.s. n.s.	hCG hCG + FSH	n.a. Varied	n.a. n.s.	hCG 1000 IU 2x/week, adjusted to TT levels	n.s. n.s.	n.s. n.s.	Weak
Giagulli HH Panhypopit [37]	2006	Prospective intervention study	Italy	6 6	36 ± 9 37 ± 9	10.5 ± 6.9 12.0 ± 3.6	n.s. n.s.	50 0	n.s. n.s.	n.s. n.s.	hCG + FSH hCG + FSH	After After	n.s. n.s.	hCG 5000 IU/week and FSH 75 IU 3x/week	24 24	24 24	Weak
Giagulli [38] Pre‐pub Post pub	2012	Non‐comparative intervention study	Italy	12 11	28 36	5 16	25 (unilateral) 0	100 0	83 n.a.	83 100	hCG + FSH hCG + FSH	Simul Simul	n.a. n.a.	hCG 5000 IU/week and FSH 75 IU/day	Max 30 Max 30	n.s. n.s.	Weak
Jones [39]	1993	Prospective cohort	UK	9	22 ± 3	1.3 ± 0.6	56 (n.s.)	78	n.s.	44	hCG + FSH	Simul	n.a.	hCG 5000—10,000 IU/week and FSH 262.5‐525/week	10.6 ± 6.8	n.s.	Weak
Kirk [40] Crypto Non‐crypto	1994	n.s.	UK	13 13	24 ± 8 22 ± 5	1.3 ± 0.5 1.8 ± 0.9	100 (54% uni‐, 46% bilateral) 0	n.s. n.s.	n.s. n.s.	77 23	Varies Varies	After After	n.s. n.s.	hCG 1000–2000 IU 2x/week or hCG 500 2x/week + hMG 300 3x/week	> 6 > 6	n.s. n.s.	Weak
Kliesch [41] IHH + kall hypopit	1994	Retrospective cohort	Germany	11 12	31 ± 7 34 ± 4	6.4 ± 2.5 15.5 ± 10	8 (n.s.) 27 (n.s.)	100 0	53 76	53 76	hCG + FSH hCG + FSH	Simul Simul	n.a. n.a.	hCG 1000–2500 IU 2x/week + hMG 150 IU 3x/week	n.s. n.s.	n.s. n.s.	Weak
Kung [42] hCG hCG + hMG	1994	Retrospective cohort	China	5 11	34 ± 3 36 ± 5	13.0 ± 7.6 2.1 ± 1.0	0 0	33 82	20 36	100 100	hCG LH + hCG	n.a. After	n.a. 6 months of azoospermia	hCG 1500 IU 2x/week and FSH 225–450 IU/week	>6 >6	n.s. n.s.	Weak
Ley [43]	1985	Retrospective cohort	USA	13	28 ± 6	2.9 ± 2.1	31 (n.s.)	62	n.s.	100	hCG + FSH	After	n.s.	hCG 1500–4000 IU 3x/week and FSH 150 IU 3x/week	18 ± 7	n.s.	Weak
Lin [44]	2019	Prospect RCT with GnRH	China	117	21 ± 8	2.4 ± 1.9	n.s.	100	0	0	hCG + FSH	Simul	n.a.	hCG 500—10,000 1‐IU 2x/week+ FSH 75–150 IU 1–2x/week	18	18	Moderate
Liu [45]	1988	Non‐comparative intervention study	USA	11	23 ± 2	2.1 ± 0.2	n.s.	100	n.s.	82	hCG + FSH	Simul	n.a.	hCG 2000 IU 3x/week and FSH 150IU 3x/week	24	24	Moderate
Liu [46]	1999	Non‐comparative intervention study	Australia	10	37 ± 7	4.6 ± 3.1	40 (n.s.)	0	n.s.	30	hCG + FSH	After	n.s.	hCG 2000 2–3x/week and FSH 150 IU 3x/week	24	24	Weak
Matsumoto [47]	2009	Non‐comparative intervention study	USA	25	n.s.	4.5 ± 1.3	4 (n.s.)	n.s.	n.s.	96	hCG + FSH	After	6 months of azoospermia	hCG 250–2500 IU/week and FSH 150–300 IU/week	18.3 ± 1.1	18	Weak
Milsom [48]	2012	Retrospective cohort	New Zea	9	38 ± 5	4.1 ± 3.0	11 (n.s.)	75	n.s.	n.s.	Varies	After	n.s.	hCG 2000 IU 2x/week and FSH 150 IU 3x/week	5.4 ± 1.9	n.s.	Weak
Miyagawa [49]	2005	Retrospective cohort	Japan	36	27 ± 9	3.8 ± 3.6	28 (n.s.)	81	n.s.	n.s.	hCG + FSH	After	n.s.	hCG 2000 IU 2x/week and FSH 2×150 IU/week	56 ± 11	12‐240	Weak
Morris [50]	2021	Non‐comparative intervention study	UK	16	37 ± 7	7.3 ± 4.6	n.s.	27	n.s.	100	hCG + FSH	After	normal T level	hCG 2000 IU 3x/week and FSH 150 IU 3x/week	14	15 ± 9	Weak
Nieschlag [51]	2017	Non‐comparative intervention study	Germany	18	32	8.6 ± 6.1	0	78	n.s.	n.s.	hCG + FSH	After	4 months normal T and persistent azoosperm	hCG 1500–3000 IU 2x/week + FSH 150 IU 2x/week	9	9	Weak
Oldereid [52]	2010	Retrospective cohort	Norway	17	n.s.	11.9 ± 7.9	12 (n.s.)	65	18	n.s.	Varies	After	3 months normal T and persistent azoosperm	hCG 2500–500 IU 2–3x/week and FSH 75–150 IU 3x/week	>24	n.s.	Weak
Ortac [53]	2019	Retrospective cohort	Turkey	135	n.s.	5.2 ± 2.4	13 (n.s.)	100	n.s.	n.s.	Varies	After	6 months normal T and persistent azoosperm	hCG 1500 IU 2x/ week and FSH 75–150 IU 2x/week	n.s.	n.s.	Weak
Resorlu [54] Pre‐pub Post‐pub	2009	Retrospective cohort	Turkey	11 6	30 27	5.7 10.2	27 (n.s.) 33 (n.s.)	100 0	64 n.a.	64 n.s.	hCG + FSH hCG + FSH	After After	3 months 3 months	hCG 5000 IU 3x/week + FSH 75 IU 1x/week	18–24 4–18	18–24 4–18	Weak
Rohayem [55]	2016	Retrospective cohort	Germany	51	n.s.	13 ± 15	n.s.	80	83	83	hCG + FSH	After	3 months	hCG 1500 IU 2x/week and FSH 150 IU 2–3x/week	n.s.	n.s.	Weak
Rohayem [56] Prior TRT No TRT	2017	Prospective comparison	Germany	34 26	16 19	4.6 ± 4.7 5¨0 ± 3.4	45 (n.s.) 32 (n.s.)	100 100	0 100	0 100	hCG + FSH hCG + FSH	After After	Normal T level 3 months	hCG 1500 IU 2–3x/week and FSH 150 IU 3x/week	24 ± 7 22 ± 6	n.s. n.s.	Strong
Saal [57]	1991	Retrospective cohort	Germany	16	23 ± 5	11.5 ± 5.5	6 (bilateral)	100	94	25	hCG + FSH	Simul or after	n.s.	hCG 3000—15,000/week and hMG from 150 uptitrated	13.3 ± 7.2	28	Weak
Samli [58]	2004	Retrospective cohort	Turkey	6	44 ± 3	1.6 ± 0.5	n.s.	n.s.	n.s.	n.s.	hCG + FSH	Simul	n.a.	hCG 200–5000 2–3x/week FSH 75–150IU 2–3x/week	21.1 ± 8.8	24	Weak
Sanyal [59] hCG hCG + FSH	2016	Retrospective cohort	India	14 5	29 ± 7 30 ± 6	3.0 3.1	n.s. n.s.	n.s. n.s.	n.s. n.s.	n.s. n.s.	hCG hCG + FSH	n.a. Simul	n.a. n.a.	hCG 2000 IU 2x/week and FSH 100 IU 2x/week	6 6	6 6	Weak
Schaison [60]	1993	Retrospective cohort	France	5	n.s.	4 ± 2	n.s.	100	n.s.	n.s.	hCG + FSH	Simul	n.a.	hCG 1500 IU/week and hMG 150 IU 2x/week	24	24	Moderate
Schopohl [10]	1991	Non‐comparative intervention study	Germany	18	24 ± 7	3.6 ± 3.0	28 (unilateral)	100	n.s.	33	hCG + FSH	After	2‐3 months	hCG 2500 IU 3x/week and FSH 150 2x/week	7.5 ± 2.8	n.s.	Moderate
Shah [61] Without TRT With TRT	2020	Retrospective cohort	India	17 18	23 ± 5 26 ± 7	4.2 ± 2.3 3.2 ± 2.3	14 (n.s.) 0	100 100	0 100	0 100	hCG + FSH hCG + FSH	Simul Simul	n.a. n.a.	hCG 2000 2x/week and FSH 75 IU 3x/week	35.2 ± 10.5 31.1 ± 14	n.s. n.s.	Weak
Singhania [62]	2024	Prospective intervention	India	16	22 ± 5	1.0	0	100	81	81	hCG + FSH	Simul	n.a.	hCG 2000 IU 3x/week and FSH 150 IU 3x/week	n.s.	18	Moderate
Sinisi [63] Hp FSH LH	2008	Two‐arm intervention prospective	Italy	9 11	19.7 ± 1.5 21.0 ± 2.4	4.6 ± 1.2 4.7 ± 1.7	n.s. n.s.	100 100	0 0	n.s. N.s.	hCG hCG + FSH	n.a. Simul	n.a. n.a.	hCG 2000 IU 2x/week and FSH 75 IU 2–3x/week	12 12	12 12	Moderate
Sinisi [64] rFSH hp FSH 2x hp FSH 3x	2010	Retrospective cohort	Italy	10 7 7	24.9 ± 1.7 24.5 ± 8.5 24.4 ± 2.6	3.7 ± 0.3 3.7 ± 0.5 4.1 ± 0.4	0 0 0	100 100 100	n.s. n.s. n.s.	n.s. n.s. n.s.	hCG + FSH hCG + FSH hCG +FSH	After After After	Normal T level	hCG 2000 IU 2x/week and FSH 75 IU 2–3x/ week	12 12 12	24 24 24	Weak
Vicari [65]	1992	Retrospective cohort	Italy	17	22 ± 1	3.8 ± 1.2	5 (unilateral)	100	54	29	Varied	If after	Normal T level	hCG 1500 IU 3x/week	6	6	Weak
Whitten [65]	2006	Retrospective cohort	UK	5	28 ± 8	4.9 ± 7.2	n.s.	80	18	60	hCG + FSH	After	n.s.	hCG 1000–2500 IU 3x/week and FSH 75–150 IU 3x/week	n.s.	3‐6 month	Weak
Yang [66] LH LH + FSH	2012	Retrospective cohort	China	84 74	18 ± 3 13 ± 3	2.0 ± 1.1 2.1 ± 1.1	n.s. n.s.	100 100	n.s. n.s.	n.s. n.s.	hCG hCG + FSH	n.a. Simul	n.a. n.a.	hCG 2000 IU 2x/week and hMG 75 IU 2x/week	> 6 > 6	> 6 > 6	Weak
Yilmazel [67]	2021	Retrospective cohort	Turkey	71	20	n.s.	n.s.	90	n.s.	n.s.	hCG + FSH	After	3‐6 months	hcG 1500 IU 2x/week and FSH 150 IU 3x/week	<18	24	Weak
Zacharin [68] LH LH + FSH	2012	Retrospective cohort	Australia	9 10	19 ± 5 20 ± 3	4.3 ± 1.9 3.4 ± 0.8	0 10 (unilateral)	100 100	n.s. n.s.	33 50	hCG hCG + FSH	n.a. After	n.a. 3 months	hCG 1500 IU 2x/week and FSH 150–300 IU 3x/week	9 9	9 9	Weak
Zhang [13] Continual Sequential	2015	Randomized controlled inferior trial	China	33 34	23 ± 5 24 ± 5	2 ± 1.9 1.8 ± 1.6	0 0	100 100	n.s. n.s.	n.s. n.s.	hCG + FSH hCG + FSH	After After	6–18 months 6–18 months	hCG 2000 IU 2x/week and FSH 75 IU 3x/week	18 18	18 18	Moderate
Zhang [69]	2019	Non‐randomized clinical study	China	18	24 ± 5	1.16 ± 0.8	0	100	11	11	hCG + FSH	After	3‐6 months	2000 IU x2/week until TT was > 10 nmol/L or orchidometer >3 mL than hMG 75 IU x3/week for 3 months, after that 3 months HCG alone, and thereafter HCG/HMG 3‐month cyclically	24	24	Moderate
Zorn [70] Pre‐pub Post‐pub	2005	Non‐comparative intervention study	Slovenia	4 2	38 ± 9 40 ± 0	6.4 ± 6 11± 5.7	17 (n.s.) 0	100 0	100 n.a.	100 100	hCG + FSH hCG + FSH	Varied Varied	2 months n.s.	hCG 3000 IU /week, after that hMG 300 IU/week. Or tese dose simultaneously	13.3 ± 5.8 2.5 ± 0.7	n.s. n.s.	Weak

Abbreviations: EPHPP, the Effective Public Health Practice Project; FSH, follicle‐stimulating hormone; hCG, human chorionic gonadotrophin; HH, Hypogonadotropic hypogonadism; IHH, idiopathic Hypogonadotropic hypogonadism; LH, luteinizing hormone; n.s., not specified; n.a., not applicable; Pre‐pub, pre‐pubertal; post‐pub, post‐pubertal; RCT, randomized controlled trial; TRT, testosterone replacement therapy; uFSH, urinary follicle stimulating hormone.

### 3.3 Risk of Bias Assessment

Utilizing the EPHPP quality assessment tool, among the 50 included studies, one study was rated as strong quality, nine studies as moderate quality, and 40 studies as weak methodological quality (Table 1). Details regarding risk of bias assessment are reported in Table 2.

**TABLE 2:** Risk of bias assessment following the Effective Public Health Practice Project (EPHPP) quality assessment tool.

Author (year)	Selection bias	Study design	Confounders	Blinding	Data collection method	Withdrawals & dropouts	Global rating
**Abbasi (2008)**	Moderate	Moderate	Weak	Moderate	Weak	Weak	**Weak**
**Bakircioglu (2007)**	Moderate	Moderate	Weak	Moderate	Weak	Weak	**Weak**
**Bouloux (2002)**	Moderate	Moderate	Weak	Weak	Weak	Weak	**Weak**
**Bouloux (2003)**	Moderate	Strong	Storng	Weak	Strong	Weak	**Weak**
**Burgues (1997)**	Moderate	Moderate	Weak	Weak	Strong	Strong	**Weak**
**Burris (1988)**	Moderate	Moderate	Weak	Moderate	Strong	Weak	**Weak**
**Carani (1999)**	Moderate	Moderate	Weak	Moderate	Strong	Weak	**Weak**
**Chen (2020)**	Moderate	Moderate	Weak	Weak	Strong	n.a.	**Weak**
**Chen (2021)**	Moderate	Moderate	Weak	Weak	Weak	n.a.	**Weak**
**Efesoy (2009)**	Moderate	Moderate	Weak	Moderate	Strong	Weak	**Weak**
**European Metrodin HP Study Group (1998)**	Moderate	Moderate	Moderate	Weak	Strong	Moderate	**Moderate**
**Finkel (1985)**	Moderate	Moderate	Weak	Moderate	Weak	Strong	**Weak**
**Fuse (1996)**	Moderate	Moderate	Weak	Weak	Weak	n.a.	**Weak**
**Giagulli (2006)**	Moderate	Moderate	Weak	Moderate	Strong	Weak	**Weak**
**Giagulli (2012)**	Moderate	Moderate	Weak	Moderate	Strong	Weak	**Weak**
**Jones (1993)**	Moderate	Moderate	Weak	Moderate	Strong	Weak	**Weak**
**Kirk (1994)**	Moderate	Moderate	Weak	Moderate	Strong	Weak	**Weak**
**Kliesch (1994)**	Moderate	Moderate	Weak	Weak	Strong	n.a.	**Weak**
**Kung (1994)**	Moderate	Moderate	Weak	Weak	Strong	n.a.	**Weak**
**Ley (1985)**	Moderate	Moderate	Weak	Moderate	Strong	Weak	**Weak**
**Lin (2019)**	Moderate	Moderate	Moderate	Moderate	Strong	Weak	**Moderate**
**Liu (1988)**	Moderate	Moderate	Weak	Moderate	Strong	Strong	**Moderate**
**Liu (1999)**	Moderate	Moderate	Weak	Weak	Strong	Moderate	**Weak**
**Matsumoto (2008)**	Strong	Moderate	Weak	Weak	Strong	Moderate	**Weak**
**Milsom (2012)**	Moderate	Moderate	Weak	Weak	Weak	n.a.	**Weak**
**Miyagawa (2005)**	Moderate	Moderate	Weak	Weak	Strong	n.a.	**Weak**
**Morris (2021)**	Moderate	Moderate	Weak	Moderate	Weak	Moderate	**Weak**
**Nieschlag (2017)**	Moderate	Moderate	Weak	Weak	Strong	Strong	**Weak**
**Oldereid (2010)**	Moderate	Moderate	Weak	Weak	Weak	n.a.	**Weak**
**Ortac (2019)**	Strong	Moderate	Weak	Weak	Strong	n.a.	**Weak**
**Resorly (2009)**	Moderate	Moderate	Weak	Weak	Strong	n.a.	**Weak**
**Rohayem (2016)**	Moderate	Moderate	Weak	Weak	Strong	n.a.	**Weak**
**Rohayem (2017)**	Moderate	Moderate	Moderate	Moderate	Strong	Moderate	**Strong**
**Saal (1991)**	Moderate	Moderate	Weak	Weak	Weak	n.a.	**Weak**
**Samli (2004)**	Moderate	Moderate	Weak	Moderate	Weak	Weak	**Weak**
**Sanyal (2016)**	Moderate	Moderate	Weak	Weak	Weak	n.a.	**Weak**
**Schaison (1993)**	Moderate	Strong	Weak	Moderate	Moderate	Strong	**Moderate**
**Schopohl (1991)**	Moderate	Moderate	Weak	Moderate	Strong	Strong	**Moderate**
**Shah (2021)**	Moderate	Moderate	Weak	Weak	Weak	n.a.	**Weak**
**Singhania (2024)**	Strong	Strong	Moderate	Weak	Strong	Strong	**Moderate**
**Sinisi (2008)**	Moderate	Moderate	Weak	Moderate	Strong	Strong	**Moderate**
**Sinisi (2010)**	Moderate	Moderate	Weak	Weak	Strong	n.a.	**Weak**
**Vicari (1992)**	Moderate	Moderate	Weak	Weak	Strong	n.a.	**Weak**
**Whitten (2006)**	Moderate	Moderate	Weak	Weak	Weak	n.a.	**Weak**
**Yang (2012)**	Moderate	Moderate	Weak	Weak	Weak	n.a.	**Weak**
**Yilmazel (2021)**	Moderate	Moderate	Weak	Weak	Weak	n.a.	**Weak**
**Zacharin (2012)**	Moderate	Moderate	Weak	Weak	Strong	n.a.	**Weak**
**Zhang (2015)**	Moderate	Strong	Moderate	Weak	Strong	Moderate	**Moderate**
**Zhang (2019)**	Strong	Moderate	Moderate	Weak	Strong	Strong	**Moderate**
**Zorn (2005)**	Moderate	Moderate	Weak	Moderate	Weak	Weak	**Weak**

Abbreviations: EPHPP, the Effective Public Health Practice Project; n.a., Not Applicable.

### 3.4 Spermatogenesis Induction

**FIGURE 2:**
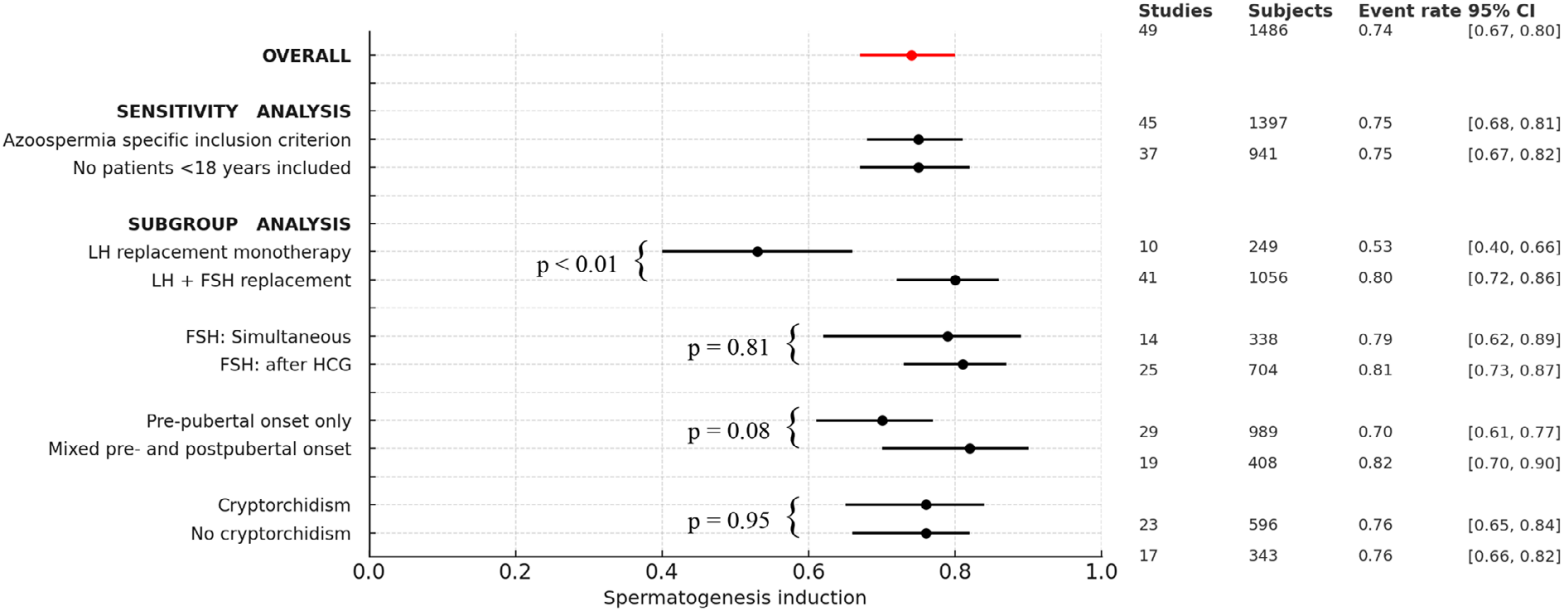
Meta‐analysis results for spermatogenesis induction of all studies, sensitivity analysis, and various subgroup analyses. Event rates of spermatogenesis induction (at least 1 spermatozoa in ejaculate) upon gonadotropin‐replacement therapy with 95% confidence intervals (CIs) are shown.

Forty‐nine studies involving 1486 male HH patients reported outcomes related to spermatogenesis induction (Figure 2). One study only reported on pregnancy outcomes [54]. Pooling of these results showed an overall success rate of 74% (95% CI: 67–80) (Figure S1). Egger's regression test indicated significant funnel plot asymmetry (*p* < 0.001), suggesting the presence of small‑study effects or publication bias. Subgroup analysis based on the type(s) of gonadotropin replacement suggested a higher success rate when both LH and FSH were replaced (80% [95% CI: 72–86] compared to LH replacement by hCG monotherapy (53% [95% CI: 40–66]) (*p* < 0.01, Figure 2). Regarding the timing of FSH addition to LH replacement therapy, no statistically significant effect on spermatogenesis induction success rate was found between simultaneous replacement or addition after hCG monotherapy (*p* = 0.81). See Table 1 for all the different addition moments for FSH. Studies only including patients with prepubertal onset HH had slightly lower rates of spermatogenesis induction compared to those including both pre‐ and post‐pubertal HH onset patients (70% vs. 82%, *p* = 0.08). Meta‑regression demonstrated a significant positive association between mean testicular volume and spermatogenesis success rate (*β* = 0.12, *p* = 0.027), with testicular volume accounting for approximately 9% of the between‑study heterogeneity (*R* ^2^ = 9.1%). Due to the limited number of studies involving TRT‐naïve patients (*n* = 3), a direct comparison with studies including patients who had previously received TRT was not feasible. Comparison between studies with and without inclusion of cryptorchidism patients revealed no difference in spermatogenesis induction success rate (*p* = 0.95).

### 3.5 Time to Spermatogenesis

**FIGURE 3:**
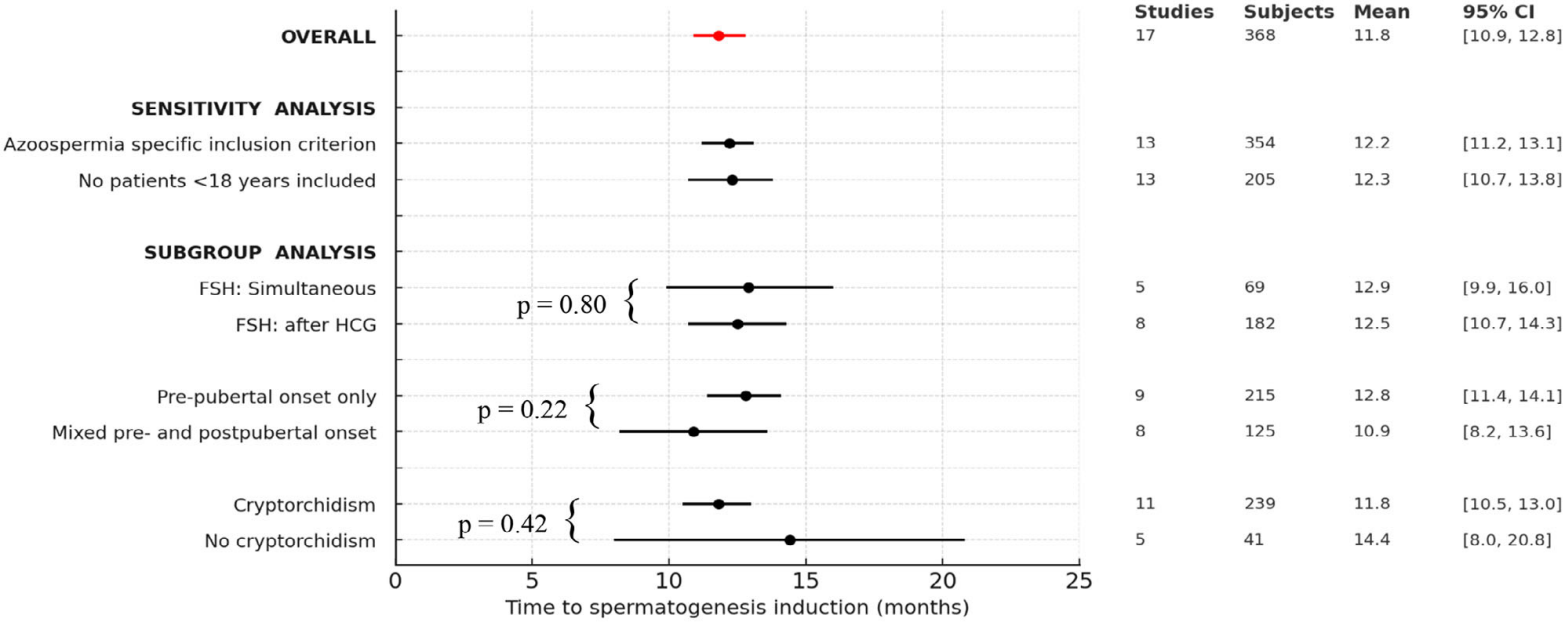
Meta‐analysis results of time to spermatogenesis induction for all studies, sensitivity analysis, and various subgroup analyses. Time to spermatogenesis induction (at least 1 spermatozoon in ejaculate) in months upon gonadotropin‐replacement therapy with 95% confidence intervals (CIs) is shown.

A total of 17 studies, including 368 males, reported on the time required to induce spermatogenesis and were included in the meta‐analysis (Figure 3). Overall, the mean time needed to induce spermatogenesis was 11.8 months (95% CI: 10.9–12.8) (Figure S2). Egger's regression test showed no evidence of funnel plot asymmetry for time to spermatogenesis (*p* = 0.24). Within the subgroup receiving replacement therapy with both hCG and FSH, no difference in time to spermatogenesis was observed when FSH was added after 1–18 months following hCG therapy (12.5 months), compared with simultaneous hCG and FSH replacement (12.9 months, *p* = 0.80). No difference was found between studies only including patients with prepubertal onset HH and those with a mixed pre‐ and post‐pubertal onset population regarding time to spermatogenesis induction (12.8 vs. 10.9 months, *p* = 0.22). Consistent with this finding, meta‑regression also did not identify a statistically significant relationship between testicular volume and the time needed to achieve spermatogenesis (*β* = ‐0.23, *p* = 0.062). No difference in time to spermatogenesis induction was found between studies, including cryptorchidism patients and study populations without cryptorchidism (*p* = 0.42). Subgroup analysis based on the type(s) of gonadotropin replacement therapy and prior TRT was not performed due to the insufficient numbers of studies per subgroup (<5 studies).

### 3.6 Sperm Concentration

**FIGURE 4:**
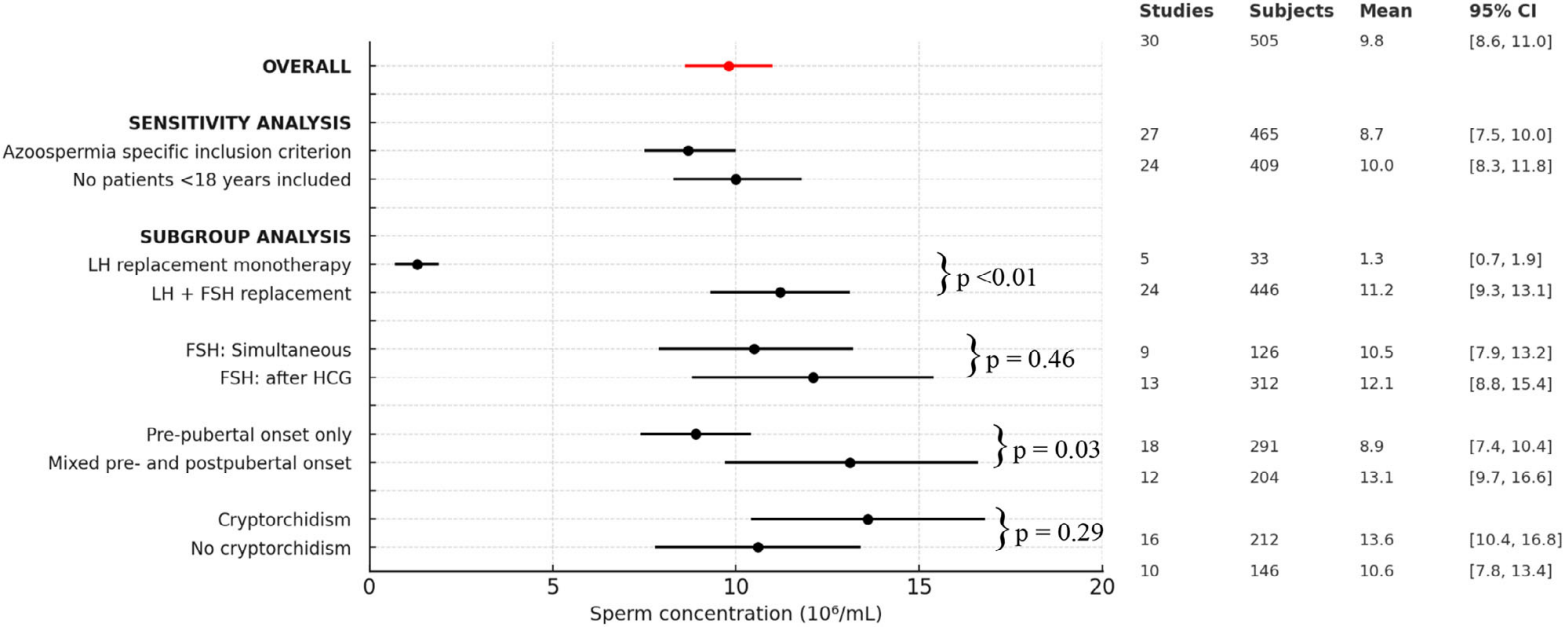
Meta‐analysis results of sperm concentration for all studies, sensitivity analysis, and various subgroup analyses. Sperm concentration in million/mL with 95% confidence intervals (CIs) is shown.

Thirty studies, involving 505 males, reported on the mean achieved sperm concentration and were included in meta‐analysis (Figure 4). The pooled analysis revealed a mean sperm concentration of 9.8 million/mL [95% CI 8.6–11.0] (Figure S3). Egger's regression test demonstrated funnel plot asymmetry for sperm concentration (*p* < 0.001), indicating strong evidence of small‑study effects or publication bias. No effect on sperm concentration was found based on timing of FSH replacement (p = 0.46) or on the inclusion of patients with cryptorchidism (*p* = 0.17). Subgroup analysis further indicated higher sperm concentrations when both LH and FSH were replaced (*p* < 0.01) and in patients with post‐pubertal onset of HH (*p* = 0.03). Meta‑regression showed no significant association between mean testicular volume and sperm concentration (*β* = 0.25, *p* = 0.10). Twenty studies, involving 396 males, provided data on the proportion of patients achieving a sperm concentration of >15 million/mL upon gonadotropin replacement therapy. The pooled results indicated that 25% (95% CI: 18–31) of all included patients reached this sperm concentration threshold (Figure S4).

### 3.7 Pregnancy Outcomes

**FIGURE 5:**
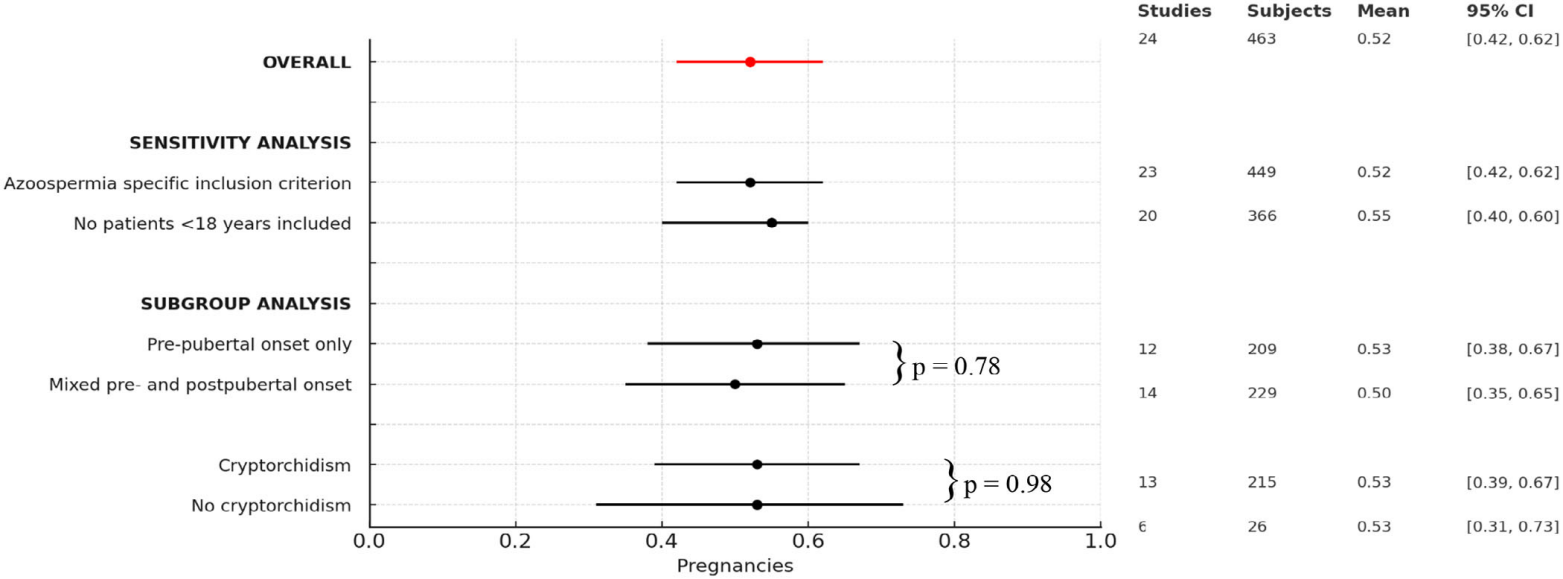
Meta‐analysis results of pregnancy success rate of all studies, sensitivity analysis, and various subgroup analyses. Event rates of pregnancy (in case of a child wish) upon gonadotropin‐replacement therapy with 95% confidence intervals (CIs) are shown.

A total of 24 studies, involving 463 males, reporting on achieved pregnancy in case of child wish were included for meta‐analysis (Figure S5). The pooled results showed a pregnancy rate of 52% (95% CI: 0.42–0.62) (Figure 5). Egger's regression test showed no significant funnel plot asymmetry for pregnancy success (*p* = 0.13). Four studies reporting mean time from treatment initiation to pregnancy showed a mean time to pregnancy of 19 months [29, 53, 55, 70]. The proportion of spontaneous pregnancy in cases of achieving pregnancy was reported in 19 studies. Meta‐analysis results showed that the pooled event rate of spontaneous pregnancy was 32% (95% CI: 23%–42%) (Figure S6). Eight studies involving 51 men reporting on the mean sperm concentration at the time of spontaneous pregnancy were included in the meta‐analysis (Figure S7). These pooled results showed that mean sperm concentration was 9.6 million/mL (95% CI 7.1–12.0) at the time of spontaneous pregnancy. In subgroup analysis according to the inclusion of patients with cryptorchidism or post‐pubertal onset of HH, no differences in pregnancy rates were found (Figure 5). Subgroup analysis according to onset of FSH addition, type(s) of gonadotropin replaced, and previous TRT was not performed due to the limited number of studies per subgroup (<5 studies).

### 3.8 Sensitivity Analyses

In an additional sensitivity analysis, studies including patients under 18 years of age or those that did not specify azoospermia as an inclusion criterion were excluded. This analysis was repeated for all outcomes (spermatogenesis induction, time to spermatogenesis, sperm concentration, and pregnancy rate), and the results remained consistent with the primary analysis. All findings continued to favor during treatment and retained statistical significance, indicating the robustness of the results to variations in inclusion criteria (Figures 2, 3, 4, 5).

### 3.9 Side Effects

Out of the 50 included studies, 11 (22%) reported side effects. Five studies found no adverse effects in their patient populations [43, 46, 50, 51, 57]. Six studies documented various side effects, with the most commonly reported being acne (11%–39%), gynecomastia (5%–33%), breast tenderness (28%), weight gain (11%), and painful injection sites (5%) [34, 47, 56, 58, 62, 69].

## 4 Discussion

This review evaluated the efficacy of gonadotropin therapy on fertility in men with HH. Our findings suggest that the combination of hCG and FSH is effective in inducing spermatogenesis and pregnancy. This study demonstrated an overall success rate of spermatogenesis induction of 74%, with a mean time to spermatogenesis of 12 months and an overall pregnancy rate of 52%, with a mean time to pregnancy of 19 months. Several systematic reviews have previously examined gonadotropin therapy in patients with HH. The most recent review (2024) focused on patients with congenital HH, assessing developmental rather than fertility outcomes [71]. Other reviews addressing adult men confirmed the efficacy of gonadotropin therapy but often included mixed‐age populations, variable endpoints, or had a narrative character [11, 12, 72]. In contrast, the present study is the first meta‐analysis dedicated exclusively to adult males with HH, providing a quantitative synthesis of fertility‐related outcomes and time‐to‐event measures.

The overall spermatogenesis induction rate of 74% observed in this meta‐analysis aligns with existing cohort studies and systematic reviews reporting rates of 64%–86% in men with HH and azoospermia [4, 11, 71]. Our subgroup analysis further showed that combined hCG and FSH therapy is superior to hCG monotherapy (80% vs. 53%, *p* < 0.01) [11, 71, 72]. In case dual therapy was performed, FSH was added 1–18 months after initiation of hCG therapy, when patients remained azoospermic with or without normalization of testosterone. This meant that, for unknown reasons, the timing of FSH addition was not always in line with the guidelines of the American Society for Reproductive Medicine and the American Urology Association, which recommends adding FSH after 3–6 months of hCG therapy, once eugonadal testosterone levels are achieved and azoospermia persists [73, 74].

In this review, a comparison between patients with or without cryptorchidism revealed no difference in spermatogenesis induction (*p* = 0.95), despite the fact that previous studies had identified cryptorchidism as a negative predictor [75, 76, 77]. Cryptorchidism was reported in 24 studies, but often in only a small proportion of the study population. Among the nine studies that specified whether cryptorchidism was unilateral or bilateral, seven included only patients with unilateral cryptorchidism. This could be an explanation for not finding cryptorchidism as a predictor of spermatogenesis. The literature consistently shows that the response to gonadotropin therapy in men with HH is worse in those with a history of bilateral cryptorchidism compared with unilateral cryptorchidism [7, 75, 78]. In men without HH, fertility outcomes are also worse in bilateral cryptorchism compared to unilateral cryptorchism [79]. Therefore, the conclusion that cryptorchidism does not affect treatment outcomes should be interpreted with caution, as data in this review on bilateral cases remain limited. Future studies should distinguish between unilateral and bilateral cryptorchidism to better clarify its impact on fertility outcomes.

The mean time to spermatogenesis was 12 months, consistent with previous literature reporting a range of 9 to 15 months for initial spermatozoa appearance following gonadotropin therapy in men with HH [71, 72]. This extended treatment duration underscores the importance of early patient counseling to set realistic expectations regarding timelines for fertility restoration. Notably, no difference in time to spermatogenesis was observed based on the timing of FSH initiation (12.5 months for sequential vs. 12.9 months for simultaneous therapy, *p* = 0.80). The timing of FSH addition varied considerably across studies; while some studies routinely introduced FSH after a fixed number of months, others added it only on indication, for instance, if normogonadal levels were reached, or in cases of persistent azoospermia. This resulted in substantial heterogeneity between study groups. Similarly, neither the onset of HH (prepubertal vs. mixed onset; 12.8 vs. 10.9 months, *p* = 0.22) nor the presence of cryptorchidism (*p* = 0.42) influenced treatment duration. These findings suggest that, while certain baseline factors may predict overall treatment success, their impact on the speed of response appears limited.

This meta‐analysis demonstrated a mean sperm concentration of 9.8 million/mL following gonadotropin therapy. This finding aligns with previous cohort studies and meta‐analyses reporting mean post‐treatment concentrations between 9 and 12 million/mL [75]. However, in this review, the majority of men do not reach normozoospermic levels according to WHO criteria, and only 25% of patients exceeded a threshold of >15 million/mL [80]. Subgroup analysis further supported the superiority of combination therapy with both hCG and FSH, which was associated with higher sperm concentrations compared to hCG replacement alone (*p* < 0.01). This aligns with clinical recommendations, such as those of the American Society for Reproductive Medicine, which endorses combined gonadotropin therapy as the standard of care for fertility induction in this population [73]. Neither the timing of FSH initiation (simultaneous vs. sequential, *p* = 0.46) nor the inclusion of patients with a history of cryptorchidism (*p* = 0.17) influenced final sperm concentration, consistent with earlier findings [75]. On the other hand, our findings showed that patients with post‐pubertal onset HH reported higher sperm concentrations than those with prepubertal onset (*p* = 0.03). This may be explained by the fact that patients with post‐pubertal onset of HH have more developed testes at the start of fertility treatment, in contrast to pre‐pubertal onset patients, where testicular development is often insufficient. This testicular underdevelopment can be treated by puberty induction. Testosterone‐induced puberty in adolescents with HH leads to virilization, without negatively impacting later spermatogenic or pregnancy outcomes following gonadotropin therapy [56, 76, 81, 82]. Puberty induction using gonadotropins or testosterone may already promote testicular growth and initiate spermatogenesis during adolescence. Some evidence suggests this could be beneficial for future fertility, but the available data are limited, and differences in outcomes are not statistically significant [81].

In our meta‐analysis, the pooled pregnancy rate was 52%. This is consistent with previously reported rates ranging from 40% to 67% in large cohorts of treated men [11, 75]. Importantly, the mean time to pregnancy was 19 months from the start of therapy. Therefore, conception may take more than a year after treatment initiation. From the moment spermatogenesis occurred, it took another seven months to pregnancy. Sperm concentrations at the time of spontaneous pregnancy remained modest, with a pooled mean of 9.6 million/mL These findings show that pregnancy was achievable even when sperm parameters remained below WHO‐defined thresholds for normozoospermia [80]. Several studies have reported successful pregnancies even with suboptimal semen parameters, spontaneous or through assisted reproductive techniques. These findings emphasize that achieving any level of spermatogenesis has meaningful clinical value, as it provides the possibility for both natural and assisted conception in men with HH [11, 32, 72]. This may be explained by relatively preserved sperm quality, particularly lower levels of DNA fragmentation, rather than by the absolute sperm count, suggesting that DNA integrity plays a critical role in fertility outcomes in this population [83]. Subgroup analysis revealed no differences in pregnancy outcomes based on cryptorchidism or post‐pubertal onset of HH. In the literature, reported predictors of successful pregnancy include larger baseline testicular volume, absence of cryptorchidism, and older age at treatment initiation, although the etiology of HH (congenital vs. acquired) does not appear to influence pregnancy rates [5, 72, 75, 84].

In this study, side effects of gonadotropin therapy were reported in only 11 of the included studies. Acne (11%–39%) and gynecomastia (5%–3%) were the most frequently reported side effects. This is comparable with previous studies, where acne, gynecomastia, and painful local injection sites were the most commonly mentioned side effects [11, 71, 85]. In these studies, the side effects were mild and reversible with dose adjustment or temporary treatment interruption [47, 56].

This review has several strengths, including a comprehensive systematic search across multiple databases, which allowed inclusion of all relevant and most recent studies. In addition, compared with previous meta‐analysis, not only spermatogenesis and pregnancy rates were assessed, but also time to event outcomes were evaluated and explored potential predictive factors across relevant patient subgroups [4, 14]. Although these positive findings, there are a few limitations for this study to consider, which may influence the robustness of the pooled estimates. (1) Study quality: the majority of included studies were of low methodological quality, with only one study rated as strong according to the EPHPP tool. The predominance of weak‐quality studies limits the confidence in the pooled estimates and underscores that the findings should be interpreted with caution. This highlights the urgent need for high‐quality, well‐designed RCTs to confirm the results. (2) Study design and heterogeneity: most studies were observational, with notable heterogeneity in study populations, interventions, and outcome definitions. This may limit the generalizability of the findings and affect the precision of pooled estimates. Residual heterogeneity may persist despite subgroup analyses and random‐effects modeling, reflecting variability in study design, reporting quality, and patient characteristics. Collectively, these sources of heterogeneity, together with indications of publication bias for some outcomes (spermatogenesis success and sperm concentration), warrant cautious interpretation of the pooled results. (3) Limited subgroup analyses: certain subgroups, such as TRT‐naïve patients or those with cryptorchidism, were underrepresented, cryptorchidism subtype was often not specified, and few studies reported outcomes stratified by pubertal induction. In addition, dosing regimens varied widely and were inconsistently reported (e.g., broad FSH ranges and patient‑specific hCG titration), precluding meaningful dose‑response or dosage‑based subgroup analyses. Consequently, the influence of these factors remains unclear. (4) Outcome measurement limitations: time to first spermatogenesis was not always consistently reported, and many studies lacked standardized hormone dosing regimens or control groups, which may introduce bias and limit generalizability. (5) Time‑to‑event reporting: time to first spermatogenesis or pregnancy was frequently reported as mean ± SD rather than median with interquartile ranges, which may inflate the reported average when a minority of patients respond only after prolonged treatment.

Despite these limitations, the findings of this review provide valuable guidance for clinical practice. The consistent benefit of combined hCG and FSH therapy in inducing spermatogenesis and achieving pregnancy supports its role as a first‐line treatment for men with HH and infertility. Future research should focus on: (1) large, well‐designed randomized controlled trials comparing different gonadotropin regimens or other treatment modalities (e.g., aromatase inhibitors, SERMs), (2) long‐term follow‐up to assess durability of fertility outcomes, hormonal stability, and potential impact on offspring health, (3) standardized reporting of outcomes, including time to spermatogenesis, live birth rates, and adverse events, and (4) consideration and detailed reporting of female partner fertility factors, such as age, reproductive history, and use of assisted reproductive techniques, to better contextualize pregnancy outcomes and minimize potential bias in assessing treatment success.

## 5 Conclusion

This systematic review and meta‐analysis demonstrate that gonadotropin therapy is effective in inducing spermatogenesis and achieving pregnancy in men with hypogonadotropic hypogonadism. In 74% of the treated men, spermatozoa were present in the ejaculate following treatment, with a mean time to spermatogenesis of 12 months, time to pregnancy from start of therapy of 19 months, and a pregnancy rate of 52%, often achieved through natural conception despite suboptimal sperm parameters. Combination therapy with both human chorionic gonadotropin and follicle‐stimulating hormone was associated with higher sperm concentrations compared with human chorionic gonadotropin monotherapy; however, evidence remains heterogeneous and largely derived from studies of moderate to low methodological quality, and thus findings should be interpreted with caution. Side effects are generally mild, with gynecomastia and acne being the most common. Although the overall evidence base is limited by methodological weaknesses and variability across studies, the collective data support gonadotropin therapy as the standard approach for fertility induction in hypogonadotropic hypogonadism. Future high‐quality studies are needed to optimize treatment protocols, identify predictive markers of success, and assess long‐term outcomes for patients and their offspring.

## Conflicts of Interest

The authors declare no conflicts of interest.

## Funding

The authors did not receive funding for this review.

## Consent

No written consent has been obtained from the patients, as there is no patient‐identifiable data included in this case report/series.

## Supporting information




**Table S1**: Full search strategy on May 15, 2025.
**Figure S1**: Cumulative event rates (with 95% CI's) of spermatogenesis induction (at least one spermatozoa in ejaculate) upon gonadotropin‐replacement therapy.
**Figure S2**: Mean time needed to induce spermatogenesis (months) with 95% CI's upon gonadotropin‐replacement therapy.
**Figure S3**: Mean sperm concentration (million/mL) with 95% CI's upon gonadotropin‐replacement therapy.
**Figure S4**: Cumulative event rates (with 95% CI's) of men achieving a sperm concentration of >15 million/mL upon gonadotropin‐replacement therapy.
**Figure S5**: Cumulative event rates (with 95% CI's) of pregnancy in couples in case of child wish upon gonadotropin‐replacement therapy. The proportion was calculated as the number of couples achieving at least one pregnancy divided by the total number of couples with a child wish.
**Figure S6**: Cumulative event rates (with 95% CI's) of spontaneous pregnancy in couples in case of pregnancy upon gonadotropin‐replacement therapy. The proportion was calculated as the number of couples having a spontaneous pregnancy divided by the total number of couples achieving pregnancy.
**Figure S7**: Mean sperm concentration (million/mL) with 95% CI's at the time of spontaneous pregnancy.

## Data Availability

Data on search results, study selection, data collection, and quality assessment are available on request.
