# Supplementary material for: Boosting Male Fertility: The Impact of Gonadotropin Therapy on Hypogonadotropic Hypogonadism—A Systematic Review and Meta‐Analysis
Source: Andrology. 2026 Apr 16;14(7):1971–87. doi: 10.1111/andr.70226 (PMC13592061; doi:10.1111/andr.70226)
Supplement: Supplementary file 1 [file ANDR-14-1971-s001.docx]

# **Supplementary material**

*Boosting Male Fertility: The Impact of Gonadotropin Therapy on Hypogonadotropic Hypogonadism - A Systematic Review and Meta-Analysis*

**M. Huijben^1*^, A.M.A. Prinsen^1*^, L.M.O. de Kort^1^, H.M.K. van Breda^1^**

^1^ Department of Urology, University Medical Centre Utrecht, Utrecht, the Netherlands

* Shared first author

**Table S1: Full search strategy on 15^th^ may 2025**

| Database | Filter | Search | Results |
| --- | --- | --- | --- |
| PubMed | **Language** (Dutch; English)  Human studies | Kallmann*[Title/Abstract] OR CHH[Title/Abstract] OR "Hypogonadotropic hypogonadis*”[Title/Abstract] OR "Hypogonadotrophic hypogonadis*"[Title/Abstract] OR "Secondary hypogonadis*"[Title/Abstract] OR Kallmann Syndrome[MeSH Terms] OR Hypogonadism[MeSH Terms] **AND** Fertil*[Title/Abstract] OR Infertil*[Title/Abstract] OR Paternity[Title/Abstract] OR "Child wish"[Title/Abstract] OR Azoospermia[Title/Abstract] OR Spermatogenesis[Title/Abstract] OR Spermiogenesis[Title/Abstract] OR Spermacytogenesis[Title/Abstract] OR Pregnanc*[Title/Abstract] OR Impregnat*[Title/Abstract] OR Infertility[MeSH Terms] OR Infertility, male[MeSH Terms] OR "fertility"[MeSH Terms] OR Paternity[MeSH Terms] OR Spermatogenesis[MeSH Terms] OR Azoospermia[MeSH Terms] OR Pregnancy[MeSH Terms] **AND** Gonadotrop*[Title/Abstract] OR HCG[Title/Abstract] OR "Chorionic Gonadotrop*"[Title/Abstract] OR Pregnyl[Title/Abstract] OR Ovitrelle[Title/Abstract] OR Choriogonin[Title/Abstract] OR FSH[Title/Abstract] OR "Follicle Stimulating Hormone"[Title/Abstract] OR "Follicle-Stimulating Hormone"[Title/Abstract] OR Follitropin[Title/Abstract] OR LH[Title/Abstract] OR Lutropin[Title/Abstract] OR "Luteinizing Hormone"[Title/Abstract] OR Menotrop*[Title/Abstract] OR Menopur[Title/Abstract] OR Gonadotropins[MeSH Terms] | 1336 |
| EMBASE | **Sources** (Embase; Embase + Medline)  **Language** (Dutch and English)  **Publication** **type** (article and article in press) | kallmann*:ti,ab,kw OR chh:ti,ab,kw OR 'hypogonadotropic hypogonadis*':ti,ab,kw OR 'hypogonadotrophic hypogonadis*':ti,ab,kw OR 'secondary hypogonadis*':ti,ab,kw OR 'kallmann syndrome'/exp OR 'kallmann syndrome' OR 'hypogonadotropic hypogonadism'/exp OR 'hypogonadotropic hypogonadism' OR 'secondary hypogonadism'/exp OR 'secondary hypogonadism' **AND** Fertil*:ti,ab,kw OR Infertil*:ti,ab,kw OR Paternity:ti,ab,kw OR ‘Child wish’:ti,ab,kw OR Azoospermia:ti,ab,kw OR Spermatogenesis:ti,ab,kw OR Spermiogenesis:ti,ab,kw OR Spermacytogenesis:ti,ab,kw OR Pregnanc*:ti,ab,kw OR Impregnat*:ti,ab,kw OR ‘Infertility therapy’/exp OR ‘male infertility’/exp OR fertility/exp OR paternity/exp OR spermatogenesis/exp OR azoospermia/exp OR pregnancy/exp **AND** gonadotrop*:ti,ab,kw OR hcg:ti,ab,kw OR 'chorionic gonadotrop*':ti,ab,kw OR pregnyl:ti,ab,kw OR ovitrelle:ti,ab,kw OR choriogonin:ti,ab,kw OR fsh:ti,ab,kw OR 'follicle stimulating hormone':ti,ab,kw OR 'follicle-stimulating hormone':ti,ab,kw OR follitropin:ti,ab,kw OR lh:ti,ab,kw OR lutropin:ti,ab,kw OR 'luteinizing hormone':ti,ab,kw OR menotrop*:ti,ab,kw OR menopur:ti,ab,kw OR 'gonadotropin'/exp OR 'chorionic gonadotropin'/exp OR 'follitropin'/exp OR 'luteinizing | 921 |
| Cochrane | Search without filters | (Kallman):ti,ab,kw OR (CHH):ti,ab,kw OR ("hypogonadotropic hypogonadism"):ti,ab,kw OR ("secondary hypogonadism"):ti,ab,kw  OR MeSH descriptor: [Kallmann Syndrome] explode all trees OR MeSH descriptor: [Hypogonadism] explode all trees **AND** (Fertil*):ti,ab,kw OR (Infertil*):ti,ab,kw OR (Paternity):ti,ab,kw OR ("Child wish"):ti,ab,kw OR (Azoospermia):ti,ab,kw OR (Spermatogenesis:ti,ab,kw) OR (Spermiogenesis):ti,ab,kw OR (Spermacytogenesis):ti,ab,kw OR (Pregnanc*):ti,ab,kw OR MeSH descriptor: [Infertility] explode all trees OR MeSH descriptor: [Infertility, Male] explode all trees OR MeSH descriptor: [Fertilizers] in all MeSH products OR MeSH descriptor: [Paternalism] in all MeSH products OR MeSH descriptor: [Spermatogenesis] explode all trees OR MeSH descriptor: [Azoospermia] explode all trees OR MeSH descriptor: [Pregnancy] explode all trees **AND** (gonadotrop*):ti,ab,kw OR (hcg):ti,ab,kw OR (“chorionic gonadotrop*”):ti,ab,kw OR (pregnyl):ti,ab,kw OR (ovitrelle):ti,ab,kw OR (choriogonin):ti,ab,kw OR (fsh):ti,ab,kw OR (“follicle stimulating hormone”):ti,ab,kw OR (“follicle-stimulating hormone”):ti,ab,kw OR (follitropin):ti,ab,kw OR (lh):ti,ab,kw OR (lutropin):ti,ab,kw OR (“luteinizing hormone”):ti,ab,kw OR (menotrop*):ti,ab,kw OR (menopur):ti,ab,kw OR MeSH descriptor: [Gonadotropins] explode all trees | 94 |

**Figure S1.** Cumulative event rates (with 95% CI’s) of spermatogenesis induction (at least 1 spermatozoa in ejaculate) upon gonadotropin-replacement therapy.

**Figure S2.** Mean time needed to induce spermatogenesis (months) with 95% CI’s upon gonadotropin-replacement therapy.

**Figure S3.** Mean sperm concentration (million/ml) with 95% CI’s upon gonadotropin-replacement therapy.

**Figure S4.** Cumulative event rates (with 95% CI’s) of men achieving a sperm concentration of >15 million/ml upon gonadotropin-replacement therapy.

**Figure S5.** Cumulative event rates (with 95% CI’s) of pregnancy in couples in case of child wish upon gonadotropin-replacement therapy. The proportion was calculated as the number of couples achieving at least 1 pregnancy divided by the total number of couples with a child wish.

**Figure S6.** Cumulative event rates (with 95% CI’s) of spontaneous pregnancy in couples in case of pregnancy upon gonadotropin-replacement therapy. The proportion was calculated as the number of couples having a spontaneous pregnancy divided by the total number of couples achieving pregnancy.

**Figure S7.** Mean sperm concentration (million/ml) with 95% CI’s at time of spontaneous pregnancy.
